# Evaluation of extracellular microRNAs as potential biomarker candidates for assessing chlamydia reinfection risk

**DOI:** 10.1371/journal.pone.0356267

**Published:** 2026-08-18

**Authors:** Chloe Meewes Lewis, Kanupriya Gupta, Howard Wiener, Hemant K. Tiwari, William M. Geisler

**Affiliations:** 1 Department of Medicine, University of Alabama at Birmingham, Birmingham, Alabama, United States of America; 2 Department of Epidemiology, University of Alabama at Birmingham, Birmingham, Alabama, United States of America; 3 Department of Biostatistics, University of Alabama at Birmingham, Birmingham, Alabama, United States of America; University of the Pacific, UNITED STATES OF AMERICA

## Abstract

*Chlamydia trachomatis* infection remains prevalent, with high rates of reinfection. Most infections are asymptomatic, and without treatment, women are at risk for reproductive and perinatal complications that are exacerbated with repeat infections. Currently, no *C. trachomatis* vaccine is available nor are there clinical biomarkers to predict reinfection risk. Small, non-coding microRNAs (miRNAs) are promising biomarker candidates because of their easy isolation, high stability, and role as molecular messengers. MiRNAs can be intracellular and/or extracellular, with the latter being released from cells and either packaged into extracellular vesicles (EVs) or freely circulating in association with proteins. We identified miRNAs from serum EVs and whole serum collected from 42 women (53 samples) and 35 women (49 samples), respectively. Sera were collected at a baseline treatment visit and a 3-month follow-up visit, where women were determined to be reinfected or not reinfected by nucleic acid amplification test. MiRNA profiles were evaluated using Bruker nCounter microarrays, data were positive ligation normalized, miRNAs were categorized as detected or not detected, nominal significance was calculated using Fisher’s exact test, and predicted target mRNAs were assessed with Qiagen’s Ingenuity Pathway Analysis (IPA). Detection of EV-derived miR-888-5p at baseline was associated with reinfection at the follow-up visit. For whole serum, detection of miR-1285-5p, −548aa + 548t-3p, and −575 at baseline were associated with absence of reinfection at follow-up. MiRs-888-5p and −548aa aligned with varying degrees to mRNA targets associated with CD8+ and CD4 + T-cell functions, with miR-888-5p demonstrating strong CD8 + T-cell associations while miR-548aa was largely associated with CD4 + T-cell responses. Distinct EV and whole serum miRNAs were identified as potential biomarker candidates that are associated with reinfection risk. Our findings are preliminary, and future work includes validation studies to confirm whether these miRNAs can predict *C. trachomatis* reinfection risk.

## Introduction

*Chlamydia trachomatis* causes the globally prevalent sexually transmitted infection, chlamydia [1]. Most infections are asymptomatic and if untreated, can potentially result in complications like pelvic inflammatory disease, infertility, chronic pelvic pain, and increased ectopic pregnancy risk [2,3]. In addition to high infection rates, reinfection is common within a year following treatment and exacerbates risk for reproductive complications [4]. In the absence of a *C. trachomatis* vaccine, improved clinical management strategies could help address these high infection and reinfection rates. One novel strategy to address this need is to utilize biomarkers to guide testing for repeat infections so that treatment can be provided before the onset of sequelae. Extracellular microRNAs (miRNAs) have emerged as promising biomarker candidates in infectious diseases [5]. These RNAs are small, non-coding posttranscriptional gene expression regulators that are specific for tissue or cell types, and their levels may vary with disease progression and response to therapy [6]. They can be present inside and/or outside a cell, the latter where they either circulate freely, are associated with proteins, or are packaged into membrane-bound extracellular vesicles (EVs) [7]. Extracellular miRNAs are ideal biomarkers because they are stable and easy to isolate from non-invasive bodily fluids like serum [6,8]. While EV isolation can be tedious, EVs offer protection from RNA degradation and are an enriched source of miRNAs compared to whole serum, which captures both EV-derived and free-floating miRNAs [9]. Although there are currently no commercially available miRNA biomarkers, findings from infectious disease research have been promising [5], especially with *Mycobacterium tuberculosis*tuberculosis, where serum miRNAs have been reported to distinguish latent versus active infection and predict risk for developing an active infection upon exposure [10,11]. With respect to chlamydia, there are limited murine and human miRNA studies [12]. Promising murine work has demonstrated miRNAs can predict pathology and exhibit dysregulation in different infection outcomes including reinfection [13,14]. Sparse human work includes one biomarker study conducted in patients with the chlamydia eye infection, trachoma, that found candidate miRNAs could not predict trachoma progression or scarring [15–17]. Additionally, two clinical genital chlamydia studies identified miRNAs associated with infection and reported distinct miRNA profiles between symptomatic and asymptomatic infections [18,19]. Thus far, no *C. trachomatis* human study has investigated miRNA biomarkers as predictors for reinfection risk; such biomarkers could be implemented in *C. trachomatis* testing strategies to improve the clinical management of chlamydia. To address this knowledge gap and determine whether miRNAs can be used to assess reinfection risk, we performed a comparative assessment of miRNA profiles from serum-derived EVs and whole serum in women treated for *C. trachomatis* infection who were then determined to have or not have reinfection at a 3-month follow-up visit.

## Materials and methods

### Cohort design and sample collection

Serum EV derived miRNAs and whole serum miRNAs were evaluated in 42 and 35 women, respectively, from a previously described study cohort of non-pregnant women presenting to the Jefferson County Department of Health (JCDH) Sexual Health Clinic in Birmingham, Alabama, USA, for treatment of a recent positive *C. trachomatis* nucleic acid amplification test (NAAT) [20,21]. Women with prior hysterectomy or antibiotic use in the past 30 days were excluded. Women were enrolled in the cohort from the 27th of March 2012 till the 11th of October 2018. At the baseline treatment visit (time of enrollment), the following were obtained: clinical information by an interview, a vaginal swab for wet mount, an endocervical swab for *C. trachomatis* and *Neisseria gonorrhea* NAAT, and blood for serum isolation. All participants were given azithromycin 1g orally as directly observed treatment, which was a first-line Center for Disease Control and Prevention (CDC) recommended chlamydia treatment at the time of the original study [22]; expedited partner therapy was not offered as it was not part of the clinic’s standard practice at that time. Participants were scheduled for a 3-month follow-up visit for another interview and for repeat collection of the same specimens and testing as was done for the baseline visit. The follow-up interview also included a question on whether the participant had been sexually active since the last study visit. All participants provided written informed consent. The study was approved by the University of Alabama at Birmingham (UAB) Institutional Review Board (IRB) and JCDH. The IRB also approved participation of minors 16 years of age or older without parental consent.

### Sample selection for miRNA testing and categorization of analysis groups

For this study, we excluded samples from miRNA testing consideration if the participant had a co-urogenital infection (gonorrhea, candidiasis, trichomoniasis, and/or bacterial vaginosis) or if there was red blood cell contamination in a serum sample, both of which could impact miRNA expression. Samples were categorized a priori into four groups for analysis purposes: (1) RB: baseline visit for those with reinfection at follow-up (*C. trachomatis* NAAT positive at both baseline and follow-up visits), (2) RF: follow-up visit for those with reinfection at follow-up, (3) NRB: baseline visit for those without reinfection at follow-up (*C. trachomatis* NAAT positive at baseline and *C. trachomatis* NAAT negative at follow-up), and (4) NRF: follow-up visit for those without reinfection at follow-up. Our primary analysis was the baseline comparison between NRB and RB, to identify potential miRNA biomarkers for further validation in additional cohorts. Secondary analyses included comparisons between (1) RB and RF to assess miRNA changes from baseline to follow-up, and (2) RF and NRF to determine differences in miRNA profiles at follow-up in women with and without *C. trachomatis* reinfection. Of 53 EV samples tested for miRNAs (collected from 42 women at baseline and/or follow-up), 24 were from the baseline visit (13 from NRB and 11 from RB) and 29 from the 3-month follow-up visit (19 from NRF and 10 from RF). For the 49 whole serum samples (collected from 35 women at baseline and/or follow-up), 26 were from baseline (14 from NRB and 12 from RB), and 23 from follow-up (15 from NRF and 8 from RF). For EV samples, 11 out of 53 were from the same women at both baseline and follow-up, while 14 of 49 whole serum samples were from the same women at baseline and follow-up. Limited sample availability, stringent sample exclusion criteria, and adequacy of miRNA yield for testing accounted for the difference in the number of samples tested for miRNA in EVs compared to whole serum and for baseline versus follow-up visits. When sample volume only allowed for miRNA testing in either EV or whole sera but not both, we prioritized EV over whole serum testing because EVs offer a more stable, specific source (vesicular-derived) for miRNAs [6–8].

### EV and RNA isolation

1 mL of serum was spun at 2,000 g to pellet any debris, and EVs were isolated using Qiagen’s exoEasy Maxi Kit (Qiagen, Hilden, Germany). Following isolation, EVs were spun in Amicon Ultra-3 KDa cutoff columns (MilliporeSigma, Burlington, MA) to remove exoEasy kit buffers. EVs were suspended in phosphate-buffered saline, and the Norgen Exosomal RNA isolation kit (Norgen Biotek Corp., Ontario, Canada) was used to isolate total RNA. Isolated RNA was cleaned and concentrated to 20 ul using Amicon Ultra-3KDa cutoff columns.

### Whole serum RNA isolation

Total RNA was isolated from 1 ml of serum using the Norgen Plasma/Serum Circulating and Exosomal RNA Purification Kit (Slurry Format) (Norgen Biotek Corp., Ontario, Canada). Isolated RNA was cleaned and concentrated to 20 ul using Amicon Ultra-3KDa cutoff columns.

### nCounter microarray

Eppendorf vacufuge (Eppendorf, Hamburg, Germany) was used to further concentrate approximately 20 ul of isolated RNA to 6 ul for serum EV RNA and 9 ul for whole serum RNA. From the final concentrated volume, 3 ul was utilized for miRNA quantification using nCounter Human v3 miRNA panels (Bruker Corp., Massachusetts, USA) that include 798 human miRNAs. nCounter Digital Analyzer, MaxFlex, detected and counted miRNAs after sample hybridization for 21 hours with nCounter Prep Station in accordance with the manufacturer’s protocol [23].

### Data processing and statistical analysis

Raw data were uploaded to nSolver version 4.0 (Bruker Corp., Billerica, MA) for quality control (QC) to assess imaging, binding density, positive controls, limit of detection, and ligation. Following QC, data set was positive ligation normalized using nSolver version 4.0 (Bruker Corp., Massachusetts, USA). To determine the appropriate detection threshold, we looked at the greatest negative control count across both datasets [24], which was 44 counts. We then set our cut-off to ≥50 counts and performed a receiver operating characteristic (ROC) curve analysis using positive (≥50) and negative (<50) values, and the area under the curve (AUC) was 0.99, indicating almost perfect distinction between positive and negative groups. Youden’s index found 49 counts to be the optimal threshold for the EV dataset and 50 counts to be the optimal threshold for the WS dataset; therefore, our cut-off was set at ≥50 for both EV and WS datasets to maximize sensitivity and specificity for differentiating positive from negative samples (S1 File) [25]. Differences in baseline participant characteristics and infection outcomes were assessed using Fisher’s exact or Wilcoxon rank-sum tests, as appropriate. Differences in the detection of specific miRNAs between the reinfection and no reinfection analysis groups were analyzed using Fisher’s exact test. Statistical correction for multiple comparisons was calculated using the Bonferroni correction ($p<6.25\times 10^{-10}$), but given the preliminary nature of this work, nominal *p*-values (*p* < 0.05) are presented. All analyses were conducted using R version 4.5.0.

### Ingenuity pathway analysis

Nominally significant baseline miRNAs and their corresponding P-values were uploaded into Qiagen’s Ingenuity Pathway Analysis (IPA) to predict their target mRNAs and identify associated biological functions. Ingenuity’s Knowledge Base served as the reference set, and the Functional Analysis tool identified biological functions and diseases most relevant to the input miRNA dataset. IPA nominal significance was calculated using right-tailed Fisher’s exact test to determine the probability of biological functions being assigned to respective mRNAs due to chance. Biological functions associated with CD4+ and CD8 + /cytotoxic T-cell responses were summarized due to their known importance in protective immunity against *C. trachomatis* [20,26–28] Unfiltered IPA biological function data is provided in full in the supplementary materials (S2-S4 Files).

## Results

### Participant characteristics

Samples tested for EV-derived miRNAs came from young women (median age = 22), in whom 98% reported African American race, none reported Hispanic ethnicity, 62% reported prior *C. trachomatis* infection, and 57% were asymptomatic (S1 Table). Similarly, samples tested for whole serum-derived miRNAs came from young women (median age = 22), in whom 97% reported African American race, 3% identified as Hispanic, 54% reported prior *C. trachomatis* infection, and 46% were asymptomatic (S1 Table). At the follow-up visit, most women reported being sexually active since the previous study visit. For EV and WS groups, all women with reinfection reported sexual activity, compared to 92% (1/13) of women without reinfection in the EV group and 86% (2/14) without reinfection in the WS group. For both EV and WS samples, baseline characteristics did not differ significantly between reinfection and no reinfection outcomes (all p≥0.05) (S5 File).

### Differential detection of EV-derived miRNAs

At the baseline visit, only one EV miRNA, miR-888-5p, had nominally significant differential detection (*p* = 0.031) (Table 1 and S1 Fig). In the follow-up visit comparison, NRF versus RF, 26 miRNAs had nominally significant differences in detection frequency; the five most nominally significant were miRs-767-5p, −1286, −495-5p, -548g-3p, and -548q (*p* = 0.009, 0.011, 0.030, 0.030, and 0.030, respectively), with all five having more frequent detection in RF (Table 1). When comparing the reinfection groups across both visits, miRs-203a-5p, -30e-3p, and 495-5p were nominally significantly detected (*p* = 0.035, 0.035, and 0.012, respectively) for RB versus RF, with detection only at follow-up (Table 1). No associations were significant after applying the Bonferroni correction and no miRNAs had nominally significant differential detection in the no reinfection group across both visits (NRB versus NRF).

**Table 1:** Differential detection of extracellular vesicle-derived MicroRNAs (miRNAs) in relation to study visit and chlamydia reinfection status.

Baseline Visit for Those Without Reinfection (NRB) vs. Reinfection (RB) at Follow-Up			
miRNA	% Detection at NRB (N = 13^a^ n (%)^b^	% Detection at RB (N = 11)^a^ n (%)^b^	*P*-value^c^
miR-888-5p	0 (0)	4 (36)	0.031
**Follow-Up Visit for Those Without Reinfection (NRF) vs. Reinfection (RF) at Follow-Up**			
**miRNA**	**% Detection at NRF (N = 19)**^**a**^ **n (%)**^**b**^	**% Detection at RF (N = 10)**^**a**^ **n (%)**^**b**^	***P*-value** ^**c**^
miR-1197	0 (0)	3 (30)	0.033
miR-1286	1 (5)	5 (50)	0.011
miR-1290e^d^	5 (26)	7 (70)	0.046
miR-144-3p	0 (0)	3 (30)	0.033
miR-184	0 (0)	3 (30)	0.033
miR-30e-5p	0 (0)	3 (30)	0.033
miR-320e^d^	16 (84)	4 (40)	0.032
miR-345-3p	0 (0)	3 (30)	0.033
miR-3605-5p	0 (0)	3 (30)	0.033
miR-378h	1 (5)	4 (40)	0.036
miR-4536-3p	1 (5)	4 (40)	0.036
miR-495-5p^e^	2 (11)	5 (50)	0.030
miR-497-5p	0 (0)	3 (30)	0.033
miR-514a-3p	0 (0)	3 (30)	0.033
miR-522-3p	1 (5)	4 (40)	0.036
miR-548aa + 548t-3p^d^	0 (0)	3 (30)	0.033
miR-548ah-5p	0 (0)	3 (30)	0.033
miR-548g-3p	2 (11)	5 (50)	0.030
miR-548q	2 (11)	5 (50)	0.030
miR-548y	1 (5)	4 (40)	0.036
miR-570-3p	0 (0)	3 (30)	0.033
miR-597-5p	0 (0)	3 (30)	0.033
miR-615-3p	0 (0)	3 (30)	0.033
miR-767-5p	0 (0)	4 (40)	0.009
miR-922	0 (0)	3 (30)	0.033
miR-942-5p	0 (0)	3 (30)	0.033
**Baseline Visit (RB) vs. Follow-Up Visit (RF) in Those With Reinfection**			
**miRNA**	**% Detection at RB (N = 11)**^**a**^ **n (%)**^**b**^	**% Detection at RF (N = 10)**^**a**^ **n (%)**^**b**^	***P*-value** ^**c**^
miR-203a-5p	0 (0)	4 (40)	0.035
miR-30e-3p	0 (0)	4 (40)	0.035
miR-495-5p^e^	0 (0)	5 (50)	0.012

^a^Percentage of detection (normalized miRNA counts ≥50 was considered detected) and total number of samples for each outcome (N).

^b^Number of samples with respective miRNA detection (n) and the percentage of detection.

^c^Statistical significance was calculated using Fisher’s exact test and no multiple comparison correction was applied. Nominal *P*-values are reported (P≤0.05).

^d^miRNAs were significant in whole serum and EV samples.

^e^Significant miRNAs were commonly detected between different comparison groups.

Note: No significant miRNAs for baseline visit NRB versus follow-up visit NRF in those without reinfection, this data was not shown in this table. MiR-548aa + 548t-3p are distinct miRNAs that share a single nCounter microarray probe due to high sequence similarity.

### Differential detection of whole serum-derived miRNAs

Nominally significant detection differences were observed for miRs-1285-5p, −548aa + 548t-3p, and −575 (*p* = 0.045, 0.032, and 0.001, respectively) at the baseline visit in NRB versus RB, with more frequent detection in NRB (Table 2 and S2 Fig); miR-548aa + 548t-3p are two separate miRNAs that were detected by a single nCounter probe due to high sequence similarity. For NRF versus RF at the follow-up visit, miRs-1246, −1290, −191-5p, -320e, −4516, and −496 were nominally significant (*p* = 0.009, 0.039, 0.027, 0.008, 0.039, and 0.026, respectively), with more frequent detection in NRF (Table 2). Within the reinfection group, miR-1246 was more frequently detected in RB than RF (*p* = 0.004), while miR-575 (*p* = 0.014) was only detected in RF and not in RB (Table 2). Similar to EVs, no associations were significant after applying the Bonferroni correction and no miRNAs had nominally significant differential detection in the no reinfection group across both visits.

**Table 2:** Differential detection of whole serum-derived MicroRNAs (miRNAs) in relation to study visit and chlamydia reinfection status.

Baseline Visit for Those Without Reinfection (NRB) vs. Reinfection (RB) at Follow-Up			
miRNA	% Detection at NRB (N = 14)^a^ n (%)^b^	% Detection at RB (N = 12)^a^ n (%)^b^	*P*-value^c^
miR-1285-5p	11 (79)	4 (33)	0.045
miR-548aa + 548t-3p^d^	12 (86)	4 (33)	0.032
miR-575^e^	9 (64)	0 (0)	0.001
**Follow-Up Visit for Those Without Reinfection (NRF) vs. Reinfection (RF) at Follow-Up**			
**miRNA**	**% Detection at NRF (N = 15)**^**a**^ **n (%)**^**b**^	**% Detection at RF (N = 8)**^**a**^ **n (%)**^**b**^	***P*-value** ^**c**^
miR-1246^e^	14 (93)	3 (38)	0.009
miR-1290^d^	11 (73)	2 (25)	0.039
miR-191-5p	10 (67)	1 (12)	0.027
miR-320e^d^	15 (100)	4 (50)	0.008
miR-4516	11 (73)	2 (25)	0.039
miR-496	13 (87)	3 (38)	0.026
**Baseline Visit (RB) vs. Follow-Up Visit (RF) in Those With Reinfection**			
**miRNA**	**% Detection at RB (N = 12)**^**a**^ **n (%)**^**b**^	**% Detection at RF (N = 8)**^**a**^ **n (%)**^**b**^	***P*-value** ^**c**^
miR-1246^e^	12 (100)	3 (38)	0.008
miR-575^e^	0 (0)	4 (50)	0.014

^a^Percentage of detection (normalized miRNA counts ≥50 was considered detected) and total number of samples for each outcome (N).

^b^Number of samples with respective miRNA detection (n) and the percentage of detection.

^c^Statistical significance was calculated using Fisher’s exact test and no multiple comparison correction was applied. Nominal *P*-values are reported (P≤0.05).

^d^miRNAs were significant in whole serum and EV samples.

^e^Significant miRNAs were commonly detected between different comparison groups.

Note: No significant miRNAs for baseline visit NRB versus follow-up visit NRF in those without reinfection, this data was not shown in this table. MiR-548aa + 548t-3p are distinct miRNAs that share a single nCounter microarray probe due to high sequence similarity.

### Associated T-cell functions based on IPA

IPA-predicted target mRNA biological functions were determined for miRs-888-5p, −1285-5p, and −548aa, which were exclusively detected in NRB versus RB samples, and thus potentially associated with reinfection risk (S2-S4 Files). IPA predicted various biological functions across all datasets, including inflammation and responses involving T and B lymphocytes (S2-S4 Files). IPA also identified several predicted target mRNA associations with both CD4+ and CD8 + T-cell functions. MiR-888-5p had more target mRNAs associated with CD8 + T-cell or cytotoxic functions (Table 3). In contrast, miR-548aa was mainly associated with CD4 + T-cell functions, including Th1 and Th17 responses (Table 3).

**Table 3:** Significant T-cell annotations for miR-888-5p and miR-548aa with corresponding target mRNAs determined by ingenuity pathway analysis.

T-cell Annotations^a^	*P*-value^b^	microRNA	mRNAs^c^
Expansion of CD8 positive memory T lymphocytes	p < 0.001	**miR-888-5p**	IL15, CD28
Expansion of effector memory RA-positive cytotoxic T cells	p < 0.001	**miR-888-5p**	IL15, CD28
Response of cytotoxic T cells	p < 0.001	**miR-888-5p**	IL15, IL1B, CD200R1
Th17 immune response	0.003	**miR-888-5p**	IL15, IL1B, IL17A, NR4A2
Proliferation of cytotoxic T cells	0.004	**miR-888-5p**	IL15, CD28, IL1B
Cell death of CD4 + T-lymphocytes	0.006	**miR-888-5p**	IL15, ID2, DCK, CARD8
Priming of cytotoxic T cells	0.01	**miR-888-5p**	CD28, IL1B
Stimulation of CD8 + T lymphocyte	0.01	**miR-888-5p**	IL15, IL1B
Expansion of CD8 + T lymphocyte	0.012	**miR-888-5p**	IL15, CD28, TRIB1
Response of CD8 + T lymphocyte	0.013	**miR-888-5p**	IL15, IL17A, PMAIP1
Function of CD8 + T lymphocyte	0.004	**miR-548aa**	AKT1, CD3G, TLR2, CD70
Quantity of CD4 + T-lymphocytes	0.007	**miR-548aa**	WNT5A, CCR1, ATM, E2F1, CACYBP, IL6, CD70, RHOA, PLP1, TP53 BP1, MPC1, SH2D1A, TFRC, TLR2
Abnormal morphology of Th1 cells	0.008	**miR-548aa**	IL6, SH2D1A
Response of Th17 cells	0.01	**miR-548aa**	IL25, IL6
Activation of CD4 + T-lymphocytes	0.012	**miR-548aa**	WNT5A, RGMA, STOML2, MPC1, IL6, TLR2

^a^T-cell annotations predicted by Ingenuity Pathway Analysis. Grouped by miRNA and ordered from most significant to least significant.

^b^Statistical significance was calculated using right-tailed Fisher’s exact test and no multiple comparison correction was applied. Nominal *P*-values are reported (P≤0.05).

^c^Messenger RNA (mRNA) targets associated with T-cell annotations for respective miRNAs.

## Discussion

This is the first study to profile human miRNAs in relation to *C. trachomatis* reinfection risk. Our main findings were from our primary analysis comparing the baseline visits in those who did or did not have reinfection at their follow-up visit. We found that detection of EV-derived miR-888-5p and whole serum miRs-1285-5p, −548aa + 548t-3p, and −575 at the baseline visit were nominally significantly associated with reinfection status at follow-up, suggesting these miRNAs should be further assessed for their potential as biomarkers for determining reinfection risk. Detection of miR-888-5p at baseline in some women with reinfection at follow-up but no detection at baseline in those without reinfection at follow-up, suggests this miRNA has the potential to serve as a biomarker for heightened reinfection risk. IPA found that some miR-888-5p predicted target mRNAs were associated with varying CD8 + T-cell functions, including priming, stimulation, response, proliferation, and expansion of cytotoxic memory T-cells. Conversely, target mRNAs only mapped to two CD4 + T-cell associations, namely Th17 responses and cell death of CD4 + T-cells. *C. trachomatis* immune protection is believed to be primarily mediated by Th1 responses, based on pivotal murine findings that have shown MHC class II restriction and IFN-γ secretion are crucial for protection [20,28–31]. Th17 responses may also play a protective role based on a murine vaccine finding that revealed multifunctional Th1/Th17 responses are linked to protection from rechallenge in vaccinated mice [26,32]. However, an older trachoma study found CD8 + T-cells confer protection when macaques are vaccinated with live, attenuated *C. trachomatis*, and a recent study from our program found that *C. trachomatis*-specific IFN-γ-producing CD8 + T-cells are associated with lower bacterial load in reinfected women [27,33]. While our IPA data are predictive and do not provide insight into specific target mRNA inhibition or activation, these CD8 + T-cell immune associations and our finding that miR-888-5p is only detected in RB suggests that miR-888-5p may induce a weak or limited Th1 and/or Th17 response and influences CD8 + T-cell immunity, thereby impacting bacterial clearance and reducing protective immunity, all of which could increase reinfection risk. Further experimental studies are required to confirm these suggested mechanistic functions. For whole serum, miRs-1285-5p, −548aa + 548t-3p, and −575 were detected more frequently at baseline in women without reinfection at follow-up versus those with refection, suggesting these miRNAs could serve as biomarkers to indicate lower risk for reinfection. IPA only identified mRNA targets and their associated biological functions for miR-548aa and not miR-548t-3p. Interestingly, IPA identified several important CD4 + T-cell associations for miR-548aa, unlike miR-888-5p. These CD4 + T-cell associations include quantity, activation, abnormal morphology of Th1 cells, and Th17 responses. These predicted T-cell functions may suggest a potential association of miR-548aa with protective immunity, hence why it is more frequently detected at baseline in those without reinfection at follow-up. IPA generated predicted biological functions for miR-1285-5p target mRNAs, however, there was no consistent pattern of T-cell associated functions comparable to miRs-548aa and −888-5p. Notably, a trachoma biomarker study found miR-1285-5p was upregulated in patients with scarring and inflammation, suggesting it could have a potential role in immunopathology [17]; however, our study found it was more frequently detected in those without reinfection, which suggests it may be implicated in immune protection in genital *C. trachomatis* infection. Furthermore, a recent human study found miR-1285-5p was one of several miRNAs downregulated in women with endometrial *C. trachomatis* infection [34]. The study noted that this set of miRNAs have been associated with targeting mRNAs that can dampen epithelial to mesenchymal transition (EMT), therefore, the downregulation of miR-1285-5p in women with endometrial *C. trachomatis* infection may promote EMT, which could cause reproductive tissue damage. Although our IPA analysis did not identify direct EMT annotations for miR-1285-5p, these findings taken together with our observation that miR-1285-5p is more frequently detected in women not reinfected with *C. trachomatis*, may suggest a protective role of miR-1285-5p as it is associated with EMT suppression, thereby maintaining epithelial integrity and reducing reproductive tissue associated damage. Importantly, our findings are categorical and future validation work should investigate differential expression of miR-1285-5p in relation to *C. trachomatis* infection outcomes. Our novel strategy of comparing EVs and whole serum miRNA profiles revealed three miRNAs (miRs-1290, -320e, and −548aa + 548t-3p) were detected for both sample types, out of which miR-320e was the most frequently detected at follow-up in women with no reinfection compared to women with reinfection. While frequently detected, miR-320e did not predict reinfection risk, thus its detection alone may not be useful from a reinfection biomarker perspective. Additionally, miR-1290 showed opposite detection patterns between EVs and whole serum, for EVs more frequent detection was observed at follow-up for women with reinfection, whereas in whole serum there was more frequent detection at follow-up for women without reinfection. This dichotomy suggests miR-1290 could be released from cells differently (either packaged into EVs or free floating) based on infection outcome. Despite these few commonalities, EV and whole serum miRNA profiles are distinct and yield unique potential biomarker candidates, and both sample inputs should be considered in future validation studies. Although miRNA detection from whole serum is easy to implement in clinical settings because of simpler RNA isolation methods, whole serum captures all miRNAs in the extracellular environment, including those freely circulating and those packaged into EVs [6]. EVs offer a more specific source of miRNAs that accurately reflect the active biological state and help protect against RNA degradation [35]. For these reasons, initial miRNA validation should include both whole serum and EVs to determine the most reproducible source. This work is limited by a small sample size due to prioritization of stringent exclusion criteria, notably excluding samples from women with co-infections that could confound miRNA findings, as well as sample availability. Additionally, we do not have follow-up data on the number of sexual partners and whether women were re-exposed to *C. trachomatis* with certainty between baseline and follow-up, however, samples were collected from a high-risk cohort of women attending an STI clinic, most of whom reported being sexually active between baseline and follow-up, irrespective of reinfection status. This suggests that almost all women were at risk for *C. trachomatis* re-exposure between baseline and follow-up visits. Our follow-up analysis between RF and NRF included women that were NAAT positive (reinfected) and NAAT negative (not reinfected), so differences in miRNA detection patterns between these groups could in part be related to the effect of active infection itself on miRNA expression. Likewise, comparing RB to RF can be difficult to interpret as both groups were NAAT positive at their respective visits, and interval miRNA profile changes during the 3-month time interval were not captured as interim samples were not collected between visits. We were also limited in the number of miRNAs that could be detected using our array-based approach rather than sequencing; however, the use of this comprehensive microarray was more suitable for samples with low RNA concentration (1mL of serum) and it can detect direct expression of 798 biologically relevant human miRNAs without amplification [36]. We did not perform real-time PCR validation as a prior study found a strong correlation between nCounter and PCR results [37]. Given our limited sample size, a stringent multiple testing correction was not applied to prevent excessive loss of power and the risk of overlooking biologically relevant signals, hence our reporting of nominal P-values. Our findings should therefore be considered hypothesis-generating, and future validation studies will include more subjects to allow for appropriate multiple testing correction. Additional validation studies should also include more paired subjects from baseline to follow-up visits, and the cellular source and target mRNAs of validated miRNAs biomarkers need to be experimentally determined to better understand their mechanistic functions. In summary, our investigation of human miRNAs in whole serum and EVs in relation to *C. trachomatis* reinfection revealed distinct miRNA expression patterns between sources, of which both yielded promising miRNAs worth further study. Following validation studies, these miRNAs have the potential to help guide chlamydia testing strategies by identifying and treating repeat chlamydia infections in a timely manner to prevent sequelae and further *C. trachomatis* transmission.

## Supporting information

S1 File Area under the curve results for background thresholding for EV and WS datasets. (XLSX)

S1 Table Background participant characteristics, stratified by sample input. (PDF)

S2 File Ingenuity pathway analysis (IPA) biological functions for miR-888-5p. (XLSX)

S3 File Ingenuity pathway analysis (IPA) biological functions for miR-1285-5p. (XLSX)

S4 File Ingenuity pathway analysis (IPA) biological functions for miR-548aa. (XLSX)

S5 File Statistical analyses between baseline characteristics and infection outcome. (XLSX)

S1 Fig Comparison of extracellular vesicle derived (EV) normalized counts of microRNA (miRNA), miR-888-5p, at the baseline visit in women who do not have reinfection at follow-up (NRB) versus those who do have reinfection (RB). Error bars represent the median and interquartile range. Greater variability is seen in the RB group at baseline (n = 11), with four samples that cross the 50-count detection threshold, whereas no NRB (n = 13) samples cross this threshold.
(DOCX)

S2 Fig Comparison of whole serum derived normalized microRNA (miRNA) counts at the baseline visit in women who do not have reinfection at follow-up (NRB) versus those who do have reinfection (RB). A. Normalized counts for miR-1285-5p in NRB (n = 14) and RB (n = 12). B. Counts for miR-548aa + 548t-3p. C. Counts for miR-575. Error bars represent the median and interquartile range. Overall, more samples in the NRB group meet the 50-count detection threshold compared to the RB group, with no counts higher than the threshold in the RB group for miR-575.
(DOCX)

S6 File Fisher’s exact results for EV and WS datasets. (XLSX)
