# Supplementary material for: Evaluation of extracellular microRNAs as potential biomarker candidates for assessing chlamydia reinfection risk
Source: PLoS One. 2026 Aug 18;21(8):e0356267. doi: 10.1371/journal.pone.0356267 (PMC13485068; doi:10.1371/journal.pone.0356267)
Supplement: Supplementary file 1 [file pone.0356267.s002.pdf]

Supplementary Table 1. Background participant characteristics, stratified by sample input

Characteristic	Extracellular vesicle (n=42)[a]	Whole serum (n=35)[a]
Age, y, median (IQR[b])	22 (3)	22 (5)
Black race, n (%)	41 (98)	34 (97)
Hispanic ethnicity, n (%)	0 (0)	1 (3)
No. sex partners in last 3mo, median (IQR[b])	1 (0)	1 (1)
Asymptomatic infection, n (%)	24 (57)	16 (46)
Prior chlamydia[c], n (%)	26 (62)	19 (54)

Date are n (%) except where indicated.
[a]Total number of women included in the study. These numbers are smaller than the total sample numbers as both baseline and follow-up samples were analyzed for some women.Due to sample availability, only 11 EV and 14 whole serum samples were matched from baseline to follow-up.
[b]Interquartile range.
[c]Prior chlamydia based on participant self reporting and/or medical record.
