# Supplementary material for: Evaluation of extracellular microRNAs as potential biomarker candidates for assessing chlamydia reinfection risk
Source: PLoS One. 2026 Aug 18;21(8):e0356267. doi: 10.1371/journal.pone.0356267 (PMC13485068; doi:10.1371/journal.pone.0356267)
Supplement: Supplementary file 2 [file pone.0356267.s007.docx]

NRB

RB

**MiR-888-5p**

**Supplementary Figure 1. Comparison of extracellular vesicle derived (EV) normalized counts of microRNA (miRNA), miR-888-5p, at the baseline visit in women who do not have reinfection at follow-up (NRB) versus those who do have reinfection (RB).** Error bars represent the median and interquartile range. Greater variability is seen in the RB group at baseline (n=11), with four samples that cross the 50-count detection threshold, whereas no NRB (n=13) samples cross this threshold.
