# Supplementary material for: Evaluation of extracellular microRNAs as potential biomarker candidates for assessing chlamydia reinfection risk
Source: PLoS One. 2026 Aug 18;21(8):e0356267. doi: 10.1371/journal.pone.0356267 (PMC13485068; doi:10.1371/journal.pone.0356267)
Supplement: Supplementary file 3 [file pone.0356267.s008.docx]

**A**

**B**

**C**

**MiR-548aa+548t-3p**

**MiR-575**

NRB

RB

NRB

RB

NRB

RB

**MiR-1285-5p**

**Supplementary Figure 2. Comparison of whole serum derived normalized microRNA (miRNA) counts at the baseline visit in women who do not have reinfection at follow-up (NRB) versus those who do have reinfection (RB).** *A.* Normalized counts for miR-1285-5p in NRB (n=14) and RB (n=12). *B.* Counts for miR-548aa+548t-3p. *C*. Counts for miR-575. Error bars represent the median and interquartile range. Overall, more samples in the NRB group meet the 50-count detection threshold compared to the RB group, with no counts higher than the threshold in the RB group for miR-575.
